# Stage-Specific Binding Profiles of Cohesin in Resting and Activated B Lymphocytes Suggest a Role for Cohesin in Immunoglobulin Class Switching and Maturation

**DOI:** 10.1371/journal.pone.0111748

**Published:** 2014-11-06

**Authors:** Gamze Günal-Sadık, Maciej Paszkowski-Rogacz, Kalaimathy Singaravelu, Andreas Beyer, Frank Buchholz, Rolf Jessberger

**Affiliations:** 1 Institute of Physiological Chemistry, Faculty of Medicine Carl Gustav Carus, Dresden University of Technology, Dresden, Germany; 2 Department of Medical Systems Biology, University Hospital and Medical Faculty Carl Gustav Carus, Dresden University of Technology, Dresden, Germany; 3 Cellular Networks and Systems Biology, Biotechnology Center, Dresden University of Technology, Dresden, Germany; 4 CECAD, Universität zu Köln, Köln, Germany; Florida State University, United States of America

## Abstract

The immunoglobulin heavy chain locus (Igh) features higher-order chromosomal interactions to facilitate stage-specific assembly of the Ig molecule. Cohesin, a ring-like protein complex required for sister chromatid cohesion, shapes chromosome architecture and chromatin interactions important for transcriptional regulation and often acts together with CTCF. Cohesin is likely involved in B cell activation and Ig class switch recombination. Hence, binding profiles of cohesin in resting mature murine splenic B lymphocytes and at two stages after cell activation were elucidated by chromatin immunoprecipitation and deep sequencing. Comparative genomic analysis revealed cohesin extensively changes its binding to transcriptional control elements after 48 h of stimulation with LPS/IL-4. Cohesin was clearly underrepresented at switch regions regardless of their activation status, suggesting that switch regions need to be cohesin-poor. Specific binding changes of cohesin at B-cell specific gene loci *Pax5* and *Blimp-1* indicate new cohesin-dependent regulatory pathways. Together with conserved cohesin/CTCF sites at the *Igh* 3′RR, a prominent cohesin/CTCF binding site was revealed near the 3′ end of Cα where PolII localizes to 3′ enhancers. Our study shows that cohesin likely regulates B cell activation and maturation, including Ig class switching.

## Introduction

Cohesin is a chromosome-associated multi-protein complex, conserved from yeast to man, is essential for sister chromatid cohesion, and is involved in DNA repair and recombination (for recent reviews on cohesin see [1], [2], [3], [4], [5], [6]. The somatic cohesin complex is composed of two members of the Structural Maintenance of Chromosomes family of proteins, SMC1 and SMC3, the kleisin RAD21, and either protein SA1 or SA2. SMC1 and SMC3 polypeptides form a V-shape heterodimer, linked at the central hinge domains of the two SMCs. The kleisin connects the ATPase heads of the two SMC proteins, thereby forming a ring-like structure, with which the SA protein associates. This ring or multiple rings connects two double-stranded DNA molecules.

In addition to its role in sister chromatid cohesion, the cohesin protein complex facilitates several kinds of chromatin interactions, some of which are cell type-specific [7], [8], [9], [10], [11]. Cohesin/CTCF co-localisation also aids transcriptional regulation and insulation [12], [13]. Cohesin regulates transcription through physical interaction with the mediator complex and juxtaposing enhancer and promoter regions during transcription [14], [15]. In T cells, Rad21 deficiency led to reduced promoter-enhancer looping at the TCRa locus associated with transcriptional changes [16].

Throughout B cell development, the immunoglobulin heavy chain (IgH) locus undergoes various conformational changes such as locus compaction to assure stage-specific assembly of Ig molecule [17], [18]. These conformational changes are facilitated by transcription factors, including YY1 and PAX5, and other chromatin-binding factors (Jhunjhunwala et al., 2008). EBF1 is another transcription factor that modulates B cell fate and is a downstream target of AID [19], [20]. Recent studies revealed that the co-localization of cohesin and CTCF at the variable *Igh* segments affects the usage of V gene segments during V(D)J recombination in pre B cells. Cohesin and CTCF facilitate long-range interactions between the V genes, the Eμ enhancer and some 3′ cohesin/CTCF binding sites [21], [22].

Mature B cells perform class switch recombination (CSR) upon antigen stimulation to diversify a constant antigen specificity into different effector functions by changing the constant region of the Ig molecule [23]. Following the introduction of double-strand breaks at switch (S) regions, located upstream of each Ig constant region, a DNA-loop is formed between the donor and acceptor S regions. The intervening DNA is excised and both ends are repaired by a mechanism involving the non-homologous end-joining pathway. Initiation of CSR requires transcription of the enzyme activation-induced deaminase (AID), which is essential for introducing DSBs at or near S regions. The *Igh* locus becomes accessible for AID when non-coding germ-line transcription (GLT) occurs at the specific S region to act as the target of CSR. GLT starts at intronic (I) promoters 5′ of the S regions. GLT requires long-range interactions between the common Eμ locus enhancer at the 5′, the respective I promoter, and the 3′ regulatory regions (3′RR) of the *Igh* locus [23].

Initiation of germline transcription, formation of double strand breaks at the switch (S) regions, and maintenance of synapsis between the donor and acceptor S regions likely require long-range chromosomal interactions within the Ig heavy chain locus [23], [24]. How these interactions are facilitated and regulated, and which factors are involved remains to be elucidated. Since cohesin topologically links two DNA molecules such as sister chromatids or intrachromosomally, cohesin is a candidate to facilitate these processes or to restrict them to the proper chromosomal regions. Initial evidence for a role of cohesin in regulating Ig class switch recombination in a B cell line was presented very recently [25].

To elucidate a potential role of cohesin in Ig class switching in primary B cells we investigated the association of cohesin before, during, and at a late stage of Ig class switching of murine splenic B cells. We used chromatin immuno precipitation (ChIP) of the cohesin subunit RAD21 followed by deep sequencing and determined binding profiles of cohesin that are stage- and region-specific.

## Results

### Cohesin associates with the Igh locus in a stage-specific manner

To obtain insights into the potential role of cohesin during CSR, we traced the differential binding of the cohesin subunit RAD21 to chromatin in purified B cells, which were left unstimulated (day 0) or were stimulated with IL-4 and LPS to induce the switch to IgG1. The B cells were analyzed at 48 h and at 96 h after induction of Ig class switching. Up to 21% of B220-positive B cells expressed IgG1 on the cell surface after 96 h of stimulation (Fig S1). At 96 h, typically app. 22% of the cells express the plasma cell marker syndecan (CD138). IgG1 positive B cells were purified and enriched by negative selection to eliminate chromatin derived from cells that did not switch after 96 h hours of stimulation (Fig. S1).

**Figure 1:**
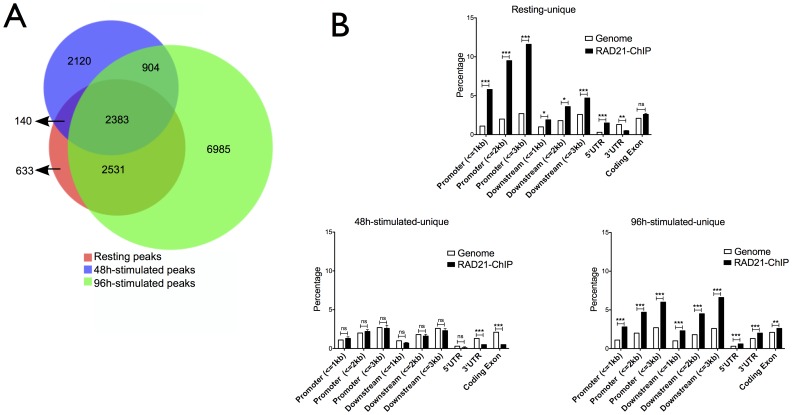
Cohesin binding sites. (A) Overlapping peaks of cohesin binding in cells with or without stimulation. Venn diagrams depict unique and common binding sites for cohesin in three different ChIP conditions based on 3 replicates, each using purified B cells pooled from 12 mice. Resting B cells: cohesin binding sites in the resting state. 48 h-stimulated B cells: binding sites detected after 48 hour stimulation of LPS and IL-4. 96 h-stimulate B cells: binding sites detected after 96 hours of stimulation with LPS and IL-4. (B) Enrichment of chromosomal features of ChIP regions. Cohesin peaks were aligned to RefSeq gene annotations by the use of CEAS tool. Each graph is based on sites unique to the indicated time point. Significance determined by one-sided binominal test. Asterisks represent p-values; ***: p<0.0001, **: p<0.001, ns: p>0.05.

Peak calling and analysis of RAD21 ChIP-Seq data revealed that cohesin binds to B cell chromatin in a stage-specific manner. Peak calling with a stringent cut-off criteria of 0.05 false discovery rate (FDR) identified 5687 cohesin binding sites in resting B cells (Fig. 1A), and 5547 binding sites in cells stimulated for 48 h with LPS and IL-4. The 96 h-stimulated B cells revealed 12803 binding sites for cohesin, implying either that cohesin binding to chromatin is more abundant in B cells stimulated for 96 h, or that the 96 h culture was more uniform, which may have resulted in a better signal-to-noise ratio and thus higher confidence peak calling. Nevertheless, the distribution of peaks clearly changed between resting, 48 h and 96 h stimulated cells.

We analyzed three biological replicates. Each replicate consisted of purified B cells pooled from 12 mice. Between all three time points 2383 sites were shared, and 140 sites were unique to the resting state. The 48 h time point yielded 2120 unique cohesin sites, and 6985 sites were only present in 96 h-stimulated cells. We conclude that cohesin occupancy significantly differs before, during and at a late stage of LPS/IL-4 induced B cell activation and Ig class switching.

### Extensive changes of cohesin binding at gene expression control regions

Since cohesin contributes to the control of gene expression and since the induction of Ig class switching involves B cell activation and specific changes in gene expression, we analyzed the cohesin binding sites specific for either time point for their potential association with transcription. Cohesin association with transcriptional elements was determined using RefSeq gene annotations through cis-regulatory element annotation system (CEAS) [26]. We compared binding of cohesin to a selected region to the average genome-wide binding.

In resting B cells, cohesin significantly (p<0.0001) binds about 4-fold more frequently to promoter regions at a distance of <1 kb, <2 kb and <3 kb from the start site than to average genomic regions (Fig. 1B). Within coding exons, however, cohesin was not enriched. Cohesin was found app. 2-fold more frequently at downstream regions of genes and app. 5-fold more frequently at 5′UTRs, but underrepresented at the 3'UTR.

At day 2 this pattern completely changed. There was no enrichment or depletion for cohesin at any of the promoter or downstream regions, nor at the 5'UTRs. Thus, upon induction of Ig class switching cohesin became redistributed to a genomic average at these loci. The underrepresentation of cohesin at 3'UTRs was maintained, but the coding exons became depleted of cohesin.

At the late 96 h time point, a cohesin pattern was re-established that largely reflected that of resting B cells. The differences were not as succinct, but cohesin was enriched app. 2-fold at promoter and downstream regions and at the 5'UTR. Cohesin was mildly enriched at coding exons. The only large difference to resting cells is in 3'UTRs, which are less bound by cohesin in resting B cells, but cohesin-enriched in B cells at day 4.

This data suggest a very significant reshuffling of cohesin at genes and their transcriptional control elements when B cells become activated and switch their Ig class.

### Cohesin is underrepresented at switch regions

**Figure 2:**
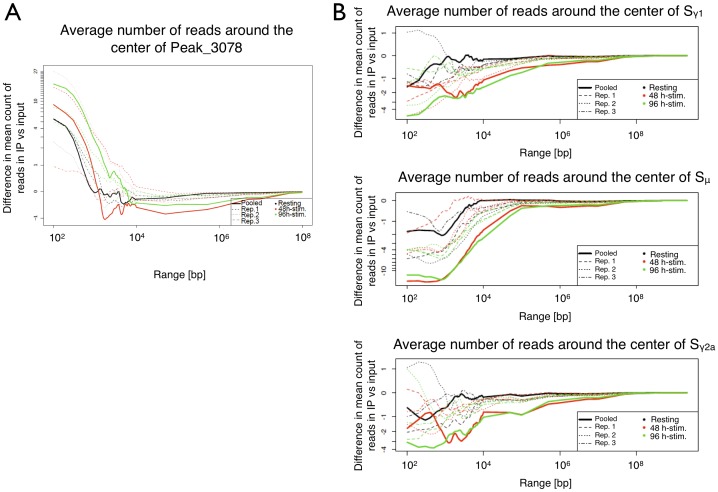
Cohesin representation at S regions. (A) A prominent MACS peak used as control (Peak 3078). (B) Counts of immunoprecipitated fragments within the S regions are plotted against the center of S regions. The coordinates of the regions under investigation: Peak 3078: chr12: 114384401-114384577; Sμ: chr12:114663654-114662317; Sγ1: chr12:114576873-114571615; Sγ2a: chr12:114530197-114533786.

It remains largely elusive how remote S regions are brought into physical proximity and how the synapsis between donor and acceptor S regions is maintained through CSR. S regions have not yet been analyzed with respect to cohesin association. To determine whether cohesin may be involved in establishment and/or maintenance of S/S synapsis, we searched for cohesin enrichment at S regions at the three time points. We measured the RAD21 reads at the centers of Sμ and Sγ1 and at increasing distance from these centers. The reads were normalized to input control. Based on this, the enrichment plots were generated to show the cohesin representation at and next to the S regions (Fig. 2). To validate the approach, a random peak was chosen to display the enrichment around this peak's center. Our analysis revealed that cohesin is under-represented for up to >10-fold at S regions in resting and at both time points after activation of class switching (Fig. 2B). At the latter two time points, cohesin was even further reduced compared to resting B cells. Similar results were obtained for the Sγ2a region, indicating that lower cohesin binding is not specific to the Sμ and Sγ1 regions. Like at Sμ and Sγ1, there was even less cohesin bound after B cell activation although Sγ2a does not undergo switch recombination. Since S regions thus feature a cohesin-poor environment, we speculate that facilitation of Sμ/Sx region synapsis by cohesin is rather unlikely.

### Cohesin binds to shared and unique sites at the Igh locus

RAD21 cohesin binds to CTCF sites in proB cells and it is likely that cohesin and CTCF facilitate V(D)J recombination by modulating long-range interactions in pro-B cells [9], [19]. To start elucidating whether cohesin may play a similar role in class switch recombination, we traced the genome wide differential binding of cohesin at the *Igh* locus.

To test the potential impact of cohesin binding on class switch recombination, in particular transcription associated to CSR, cohesin binding sites at the *Igh* locus were investigated and potential co-binding of cohesin with CTCF, PolII and B cell transcription factors PAX5 and EBF1 analyzed [20], [27], [28].

Publicly available ChIP-Seq raw data were processed and used for comparison. We first analyzed differential binding of cohesin, CTCF in *Rag1^-/-^* pro-B cells and EBF1 in resting splenic mature B cells (Table S1).

Our analysis revealed that the *Igh* locus enhancer Eμ, which is shown to be in physical proximity of the 3′RR region, is bound by cohesin neither in pro-B cells nor in mature B cells (Fig. S2) [21]. CTCF is enriched (please consider the scale, adjusted to the very strong 3′ CTCF signals) at the Eμ enhancer in mature B cells, implying that the Eμ-3′RR interaction might be facilitated via CTCF at these sites but not by cohesin binding, suggesting a cohesin-independent action of CTCF. Co-localization of EBF1 and PolII at Eμ provides further evidence for Eμ enhancer activity in mature B cells.

Since frequent cohesin binding was observed at the 3'RR (Fig. S2), we analyzed this in more detail. The 3′ end of the murine *Igh* locus contains four quasi-palindromic DNAseI hypersensitive sites (HS3a, HS1,2, HS3b and HS4) that are required for CSR [29]. Downstream of HS1-4 localizes another subset of hypersensitive sites (HS5-7) that possess conserved, overlapping CTCF and cohesin binding sites [30]. The deletion of HS5-7 region had no effect on CSR [31].

**Figure 3:**
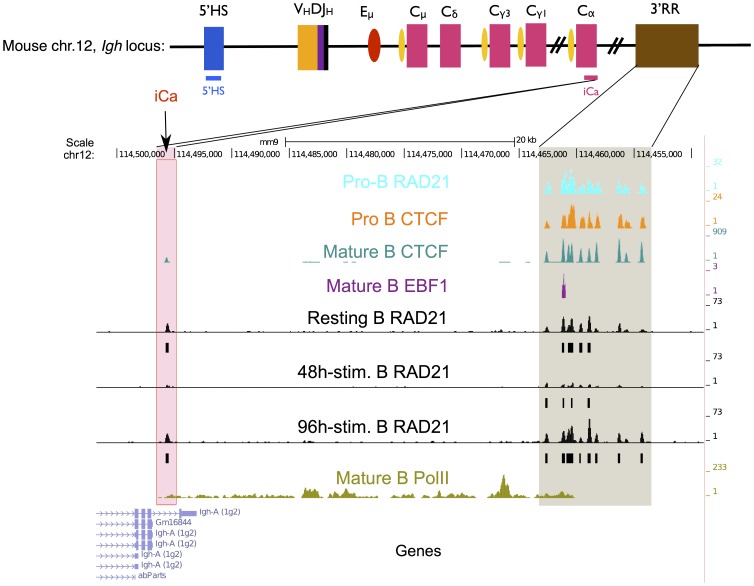
Shared and common binding events at the Igh locus. A stage-specific cohesin/CTCF site (iCa) is shown together with 3′ RR cohesin/CTCF sites.

In addition to the common cohesin/CTCF sites at the 3′ end of the *Igh* locus, we identified a rather novel cohesin/CTCF site towards the 3′ end of the intronic α constant region (intronic Cα-iCα), which overlaps with the initiation of PolII enrichment in activated mature B cells (Fig. 3). This site was only very recently described in an activated B cell line [25]. PolII enrichment indicates the active transcription through HS1-4 DNAseI hypersensitive sites and the novel cohesin/CTCF site marks the initiation of this transcription. We hypothesize that this stage-specific cohesin/CTCF site is likely to be necessary for the transcription of 3′ enhancer units that are critical for class switch recombination. Transcription factors EBF1 and PAX5 bind to HS5-7 only at one site, 3RR2 (Fig. 3 and Fig. S3).

In addition to 3′ cohesin binding events, a prominent cohesin binding site (5′HS) at the 5′ flanking region of *Igh* is observed (Fig. S4). This site corresponds to a hypersensitive site located in an intronic region of the neighboring 5′ gene, *Zfp386* [32]. This 5′HS is bound by CTCF in pro- and mature B cells, but cohesin binding is present only in mature B cells. Cohesin is diminished at day 2 when the B cells undergo class switching and re-appears at day 4. EBF1 is not present at this site, although RNA PolII shows a moderate peak. Although an *Igh*-regulatory role has previously been suggested for this HS site, the site was shown to have no effect on CSR or on VDJ recombination [32], [33].

### Changes of cohesin binding patterns at B cell-relevant gene loci

Since cohesin regulates transcription we also analyzed stage-specific cohesin binding changes at gene loci of particular importance for B cell biology.

Binding changes of cohesin at the *Aicda* locus are of interest with respect to a potential role of cohesin in modulation of CSR, since stage-specific expression of AID is indispensible for CSR. We determined shared CTCF/cohesin sites (Fig. S5) that are present in the first intron (site a), the last exon (site c) and in the second intron (site b) of the AID gene. The first intron (site a) contains low amounts of CTCF and cohesin, but it is specific to mature B cells. However, sites a and b were not detected as statistically significant cohesin peaks with the applied 0.05 FDR cut-off, although at a slightly less stringent cut-off they were. The CTCF enrichments at those sites were significant (Fig. S5). Cohesin was not enriched at the *Aicda* gene in 48 h-stimulated B cells.

**Figure 4:**
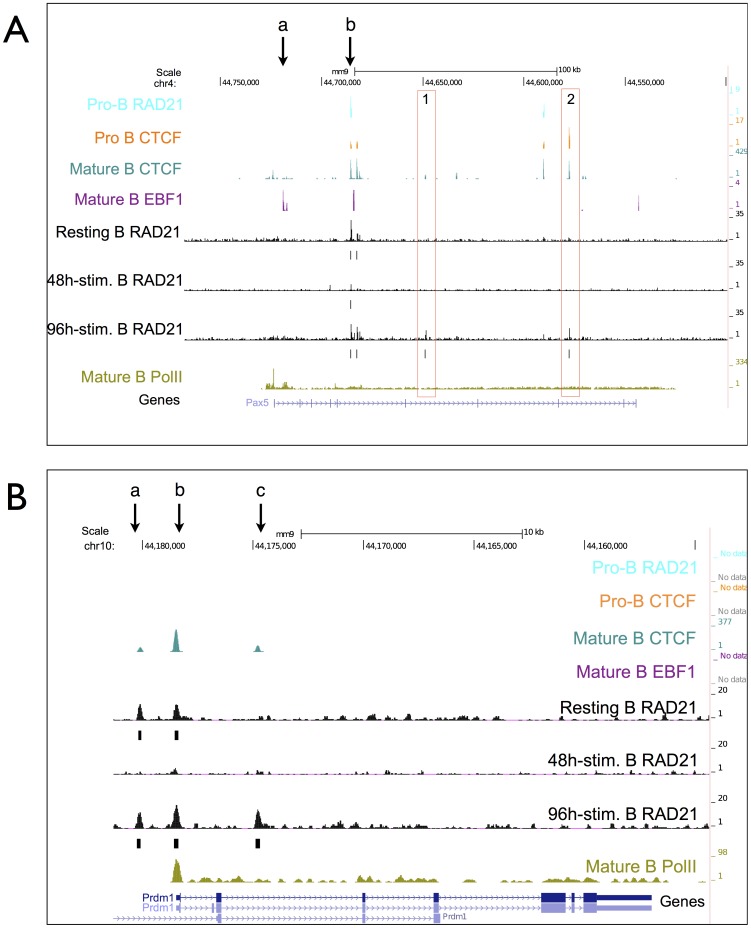
Binding profiles at the Pax5 and Prdm1 gene loci. (A) Binding profiles at the Pax5 gene locus. ChIP-seq profiles of RAD21, CTCF, EBF1 and PolII binding at the Pax5 gene. (B) Binding profiles at the Prdm1 gene locus - ChIP-seq profiles of RAD21, CTCF, EBF1 and PolII binding at the Blimp-1 gene.

The expression of B cell master regulator PAX5 is diminished as B cells differentiate into plasma cells upon completion of class switching [34]. Therefore, we investigated differential binding of cohesin at the *Pax5* gene locus (Fig. 4A).

Correlation of CTCF, cohesin and EBF1 binding is often observed at sites of PolII enrichment (Fig. 4A and data not shown). One EBF1 binding site, site b, is enriched by cohesin and CTCF both in pro- and and mature B cells, whereas the upstream EBF1 site, site a, is enriched only for CTCF and only in mature B cells. This site corresponds to the TSS with high levels of PolII. These two EBF1 sites (a and b) are suggestive of a promoter-enhancer interaction since site b is identified as a cis-regulatory site important for B-cell specific Pax5 expression [35]. Cohesin binding at site number c appears only in switched B cells and is intronic. This site is also bound by CTCF. Site number d represents a site conserved for CTCF, but not for cohesin. Cohesin binding at this site is observed only after 96 hour of stimulation. Those two cohesin binding events might suggest a role for turning off *Pax5* gene expression as B cells express IgG1 on the cell surface and become plasma cells.

The transition of splenic mature B cells into plasma cells is modulated by the transcription factor BLIMP-1 (B lymphocyte-induced maturation protein-1). BLIMP-1 is expressed in plasma cells and in a subset of germinal center B cells [36]. Forced expression of Blimp-1 drives mature B cells to plasma cell stage [37]. Therefore, we addressed binding changes of cohesin at the Blimp-1 gene, *Prdm1*, upon stimulation of the cells. The *Prdm1* promoter contains a binding site for CTCF and cohesin specific for mature B cells (Fig. 4B, site b). Around 2 kb upstream of the promoter another cohesin/CTCF site specific for mature B cells (Fig. 4B, site a) is present. At both sites, cohesin binding is reduced 48 h after initiation of stimulation. Moreover, cohesin binds frequently to a CTCF site (Fig. 4B, site c) in the second intron of *Prdm1* gene only after 96 hours of stimulation. Binding kinetics of cohesin at this CTCF site might be critical for higher *Prdm1* expression levels necessary for plasma cell identity. We conclude that cohesin binds to *Pax5* and *Prdm1* gene loci in a stage-specific manner and therefore cohesin is likely to regulate chromosomal interactions for the regulation of expression of these genes.

## Discussion

Given that cohesin regulates distinct molecular events, i.e. transcription and recombination, we directed our analysis to identify cohesin's potential roles in late B cell maturation and B cell class switch recombination and used primary mouse splenic B cells for these analyses. By performing ChIP-seq, we revealed that cohesin is enriched at sites of transcription in resting and 96 h-switch-stimulated B cells, but not in 48 h-stimulated B cells. Cohesin association to transcriptional sites implies that cohesin acts in regulation of transcription, probably by facilitation of promoter-enhancer interactions and/or RNA polII binding to those sites. Upon stimulation for 48 h, cohesin associated less with transcriptional elements. Such a pattern could be explained by the fact that during class switch recombination cells undergo several rounds of division. Presence of 5547 high-confidence cohesin binding sites in 48 h-stimulated B cells indicates that cohesin binding greatly changes upon stimulation for CSR. Since at this time point of stimulation the cells are highly activated and undergo extensive of proliferation, an overall reduction of heterochromatin and of cohesin binding to chromatin may be expected.

Our analysis reveals that cohesin is much underrepresented at S regions. Cohesin is less represented at S regions than in the flanking regions, contrary to a recent finding that suggests cohesin is associated with Sμ, although not Sγ1, upon switch-activation [25]. The difference may be explained in several ways. We have performed a more detailed analysis of the S and S-flanking regions. Our strategy of having biological replicates, which are then pooled at the stage of mapping the reads to the mouse genome, boosts signal to noise ratio and consequently increases predictive power of the peak calling algorithm. Claudepierre et al did not apply such a strategy. The use of different antibodies may also contribute somewhat to the difference. Claudepierre et al. have used an anti SMC3 antibody and thus an antibody recognizing the core SMC heterodimer of cohesin. We have used an anti RAD21 antibody and thus have targeted the much more labile kleisin subunit of cohesin, and thus those cohesin complexes that are truly bound to chromatin as fully intact cohesin complexes.

Our findings implicate that the cohesin-free nature of S regions is not specific for those S regions undergoing recombination but also seen at S regions that are not made accessible to recombination by GLT. If this is true, besides other processes like mis-regulation of AID expression, also the largely cohesin-free status of S regions may contribute to the high levels of oncogenic translocations occurring at the *Igh* locus [38]. Although cohesin is targeted to silenced chromatin in yeast, cohesin is not enriched at sites of convergent transcription in mammals [7], [39]. Therefore such underrepresentation of cohesin as seen in S regions could be distinct from other known functions of cohesin in gene regulation. Knock-down of cohesin subunits in the CH12 B cell line impaired CSR but not S region transcription, implying that cohesin plays a more global role in modulation of CSR [25]. Additionally, B cells derived from Cornelia de Lange Syndrome patients show increased microhomology-based end joining during CSR, suggesting that cohesin, and its loading factor NIPBL, are necessary for non-homologous end joining during CSR [40].

Our findings demonstrate that the iCa binding site is only enriched by cohesin and CTCF, once the cells reach mature B cell stage. iCa contains a CTCF-binding motif and PolII binding is initiated there only in mature, activated B cells. PolII is enriched at the genomic region between iCa and 3′RR CTCF/cohesin binding sites. Between the last exon of Cα and 3′ CTCF/cohesin sites, a set of DNAse hypersensitive area act as enhancers for class switch recombination (HS3a, HS1,2, HS3b and HS4). Combined deletion of these enhancers blocks switching to all isotypes [41]. Given that cohesin sits at the 3′ Cα site only at the mature stage, and that this site overlaps to the region where PolII enrichment starts, an important role of cohesin/CTCF interplay for the control of 3′ enhancer activity could be anticipated. Molecular approaches to snap-shot chromatin interactions at a given time, i.e. chromosome conformation capture, could provide further evidence for cohesin's impact in the regulation of 3′ enhancer transcription which is indispensable for switch recombination.

We did not detect cohesin/CTCF binding at the Blimp1 (*Prdm1*) gene in proB cells. The *Prdm1* promoter is bound by cohesin and CTCF only in mature B cells. Interestingly, one site of intronic cohesin/CTCF enrichment is observed only after 96 h of stimulation. One can assume that this site is only bound by cohesin as Blimp1 expression increases to drive switched B cells to become plasma cells, i.e. CD138^+^ cells, which are present at the 96 h time point. We also revealed two stage-specific cohesin/CTCF binding events at the *Pax5* gene body activated only after 96 h of stimulation, implying these a role for the termination of PAX5 transcription, which is necessary for plasma cell differentiation. One of these cohesin sites coincides with a conserved CTCF site. However, the second cohesin site is enriched for CTCF only in mature, activated B cells. Possible interactions, including those regions at the *Pax5* locus should be further addressed to clarify a potential role for these sites in diminishing PAX5 expression upon plasma cell differentiation.

Together, these data indicate potential candidate regions of regulation, suggested by cohesin binding changes upon switch-stimulation. As patients with mutations in cohesin loading factor NIPBL reveal high levels of Ig isotype deficiency [42], learning about the stage-specific binding events at the *Igh* locus and at B-cell related genes, i.e. *Pax5* and *Prdm1* will open new avenues to understanding how cohesin contributes to regulation of B cell maturation, isotype switching and associated events.

## Materials and Methods

### Cell preparation and culture conditions

C57BL/6J wild-type (wt) mice were obtained from the Jackson Laboratories and maintained under pathogen-free conditions in the animal facility of the Medical Faculty Carl Gustav Carus. The experiments were performed under approval by the Animal Welfare Committee of the State of Saxony (permission number 24-9168.24-1/2010/25). Mice were used for tissue removal only under euthanasia conditions approved by the Animal Welfare Committee of the Technische Universität Dresden. Euthanasia was performed using carbon dioxide anesthesia. Splenic B cells were isolated from 8- to 10-week-old C57BL/6J mice. The spleen was dissected from the mouse, meshed on a 40 µm nylon mesh and resuspended in culture medium Hybridoma-SFM (12045-084, InVitrogen), supplemented with 10% FCS, 100 U/ml penicillin, 100 U/ml streptomycin and 5×10^−5^ M ß-mercaptoethanol. B cell cultures were prepared using B cells purified on MACS columns according to the manufacturers instructions (130-090-862, Miltenyi Biotec). For the switch to IgG1, purified B cells were cultured at a cell density of 3×10^5^ cells/ml in complete culture medium supplemented with 50 µg/ml LPS (L6511, Sigma) and 2 ng/ml mouse recombinant IL-4 (404ML, R&D Systems).

### Flow cytometry

B cells were washed with PBS supplemented with 2 mM EDTA and 0.5% BSA followed by staining with anti-mouse B220 antibody conjugated with Pacific Blue (103230, BioLegend) and anti-mouse IgG1 antibody conjugated with APC (406609, BioLegend). Stained cells were incubated for 20 minutes on ice and were washed twice. Cell surface staining measurements were carried out with BD LSR II Flow Cytometer and data plots were generated using FlowJo software.

### Quantitative Real-Time PCR

Conventional polymerase chain reactions (PCRs) were performed using Dream Taq DNA polymerase and buffers according to manufacturer's instructions (EP0713, Fermentas). Quantitative PCRs were performed using the Rotor Gene SYBR Green PCR kit supplied from Qiagen (204074) in a Rotor Gene 3000 real-time PCR cycler, and comparative quantification analyses of PCR products and melt curve analyses were carried out using the Rotor Gene software.

### Chromatin immunoprecipitation (ChIP) and deep sequencing

B cells were cross-linked with 1% formaldehyde at room temperature for 10 minutes and cross-linking was quenched by 125 mM glycine. Cells were lysed in SDS lysis buffer (1% SDS, 10 mM EDTA, 50 mM Tris-HCl, pH 8.1) and the chromatin was sonicated to an average fragment length of 500 base pairs (bp) using a Branson Sonifier 450. Sonicated chromatin was pre-cleared and incubated with 2 µg of anti-RAD21 antibody (ab992, Abcam), 4 µl anti-CTCF (07729, Millipore), 2 µg anti-PAX5 (sc-1974, Santa Cruz) and 1 µg anti-IgG (sc-2027, Santa Cruz) overnight. The complexes were washed with increasing salt stringency and cross-links were reversed in the presence of 0.2 M NaCl at 65°C for 6 hours. Proteinase K treatment was carried out in the presence of 10 µM EDTA, 40 µM Tris-HCl, pH 6.5 and 20 µg Proteinase K. DNA fragments were recovered with phenol-chloroform extraction following one chloroform extraction. DNA was precipitated with 2 volumes of ethanol, 1/3 volumes of 7.5 M ammonium acetate and 20 µg glycogen with an overnight incubation at −20°C. Precipitated DNA was washed with 70% Ethanol to remove excess salt and DNA was resuspended in 30 µl TE buffer supplemented with 0.3 µg RNAseA. A ChIP-Seq library was prepared by the Deep Sequencing Facility of the Biotechnology Center/SFB655 of Dresden University of Technology. Immunoprecipitated fragments were end-repaired with NEBnext End Repair Module (NEB) and purified by using Agencourt Ampure XP - beads (Beckman Coulter) and A-tailed using the NEBnext dA-Tailing Module according to manufacturers' instructions. After purification, adaptors were ligated (Adaptor-Oligo 1: 5'ACACTCTTTCCCTACACGACGCTCTTCCGATCT-3', Adaptor-Oligo 2: 5'-P-GATCGGAAGAGCACACGTCTGAACTCCAGTCAC-3') by using 1x NEBnext Quick Ligation Buffer (NEB), 10x excess of DNA Adaptors, 5 µl Quick T4 DNA Ligase (NEB) at 50 µl total volume. Large-scale amplification of library constructs was performed by using the PCR Enrich Adaptor Ligated cDNA Library module (NEB). Fragment length selection was carried out by agarose gel electrophoresis and concentrations were determined by using Qubit dsDNA HS Assay Kit (Invitrogen).

### Genome-wide analyses of RAD21 ChIP-seq

Alignment of RAD21 ChIP-Seq reads to reference mouse genome assembly mm9 was performed using the Bowtie 2 version 0.12.8 (Langmead et al. 2009) with the "–very-sensitive" option. From each set of aligned reads, a subset of 7 million unique positions was randomly selected for further processing and, additionally, IP and control samples from biological replicates were merged to form additional "pooled" samples. Binding site detection in each set of biological replicates and pooled samples was done with the MACS 2 peak calling software (Yong Zhang et al. 2008) with 0.05 FDR used as a cut-off value, and with standard parameters for shifting model calculations. Binding site overlaps between different sample sets were calculated with BEDTools utilities (Quinlan & Hall 2010). Genome-wide analysis of enrichment of chromosomal features and chromosomal distribution of ChIP regions were determined using CEAS package (Shin et al. 2009) provided by Galaxy/Cistrome platform http://cistrome.org/ap (Giardine et al. 2005). Genome mapping of RAD21 binding regions and publicly available ChIP-seq data were visualized using the University of California Santa Cruz (UCSC) Genome Browser interface (Kent et al. 2002).

The GEO accession number is GSE61443 and can be accessed at http://www.ncbi.nlm.nih.gov/geo/query/acc.cgi?token=glwdcwgcrjgfzid&acc=GSE61443

### ChIP-Seq data comparison

All the ChIP-seq data listed in Table S1 was processed using the following computational pipeline. Adapter sequences, quality of raw reads, sequence duplication levels and over-represented sequences were analyzed using FastQC. Adapter sequences were removed and reads were trimmed using the FastX Toolkit (Patel & Jain 2012) such that the quality criteria of quality value per base (Phred score) was at least 35 and the read length was at least 17 bp. Raw reads were mapped to the mouse genome (mm9), using Bowtie 2 version 0.12.8 (Langmead et al. 2009) with 17 bp seed length with a maximum of two allowed mismatches. SAMtools (H. Li et al. 2009) was used to create BAM files. Peak calling was done using MACS (Yong Zhang et al. 2008) for transcription factors with 0.0001 *p*-value cutoff. Qeseq (Micsinai et al. 2012) was used for PolII binding data with 0.001 *p*-value cutoff.

## Supporting Information

Figure S1 (A) Scheme of purification of IgG1-positive and -negative B cells. (B) FACS profiles of resting B cells and B cells stimulated for 48 or 96 h. At 48 h, 2.7% of the cells were IgG1-positive. The IgG1-positive and -negative fractions are shown for the 96 h time point, where the IgG1-negative fraction contained 0.5% IgG1-positive cells, and the IgG1-positive fraction contained 65%. Many cells in the IgG1-negative gate of this fraction also started to express IgG1 as they shifted in fluorescence towards the IgG1-positive gate.
(TIF)

Figure S2 **ChIP-Seq profile of binding factors around the Eμ and 3′RR.** Binding profile of RAD21, CTCF, EBF1 and PolII in pro- and/or in mature B cells. The highest enrichment of PolII marks the locus enhancer Eμ. The highest cohesin-CTCF colocalization marks the cohesin/CTCF bound to 3′RR. Genes depict UCSC Gene annotations. ChIPSeq data plots are generated by using data obtained in this study and from publicly available sources mentioned in Table S1.
(TIF)

Figure S3 **Binding of factors at Igh locus cohesin sites.** (A) Scheme of the Igh locus binding events under investigation. (B) Enrichment of RAD21, CTCF, and PAX5 at cohesin binding sites in resting B cells were quantified by ChIP-qPCR.
(TIF)

Figure S4 **Mature B cell-specific cohesin/CTCF site corresponds to the 5′ flanking gene of Igh locus.** ChIP-Seq data plot indicates a mature B cell specific binding of cohesin subunit RAD21 at the HS region located at the 5′ of *Igh* locus.
(TIF)

Figure S5 **Binding profile at the Aicda gene locus.** ChIP-seq profiles of RAD21, CTCF, EBF1 and PolII binding at *Aicda*.
(TIF)

Table S1 **ChIP-seq data sets used for comparison studies.**
(PDF)
