# Supplementary figures and images for: Stage-Specific Binding Profiles of Cohesin in Resting and Activated B Lymphocytes Suggest a Role for Cohesin in Immunoglobulin Class Switching and Maturation
Source: PLoS One. 2014 Nov 6;9(11):e111748. doi: 10.1371/journal.pone.0111748 (PMC4222939; doi:10.1371/journal.pone.0111748)

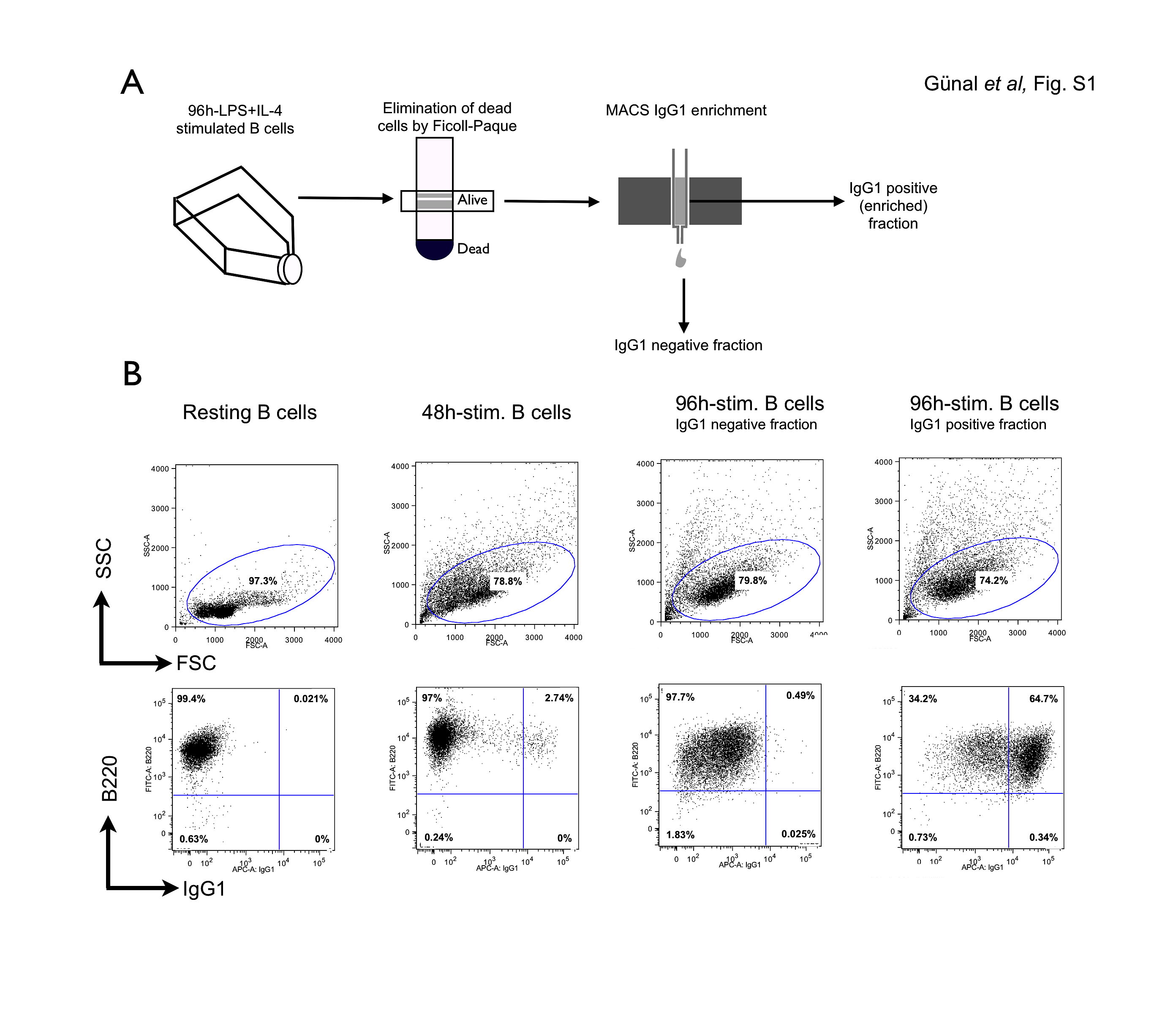

Supplement: Figure S1 — (A) Scheme of purification of IgG1-positive and -negative B cells. (B) FACS profiles of resting B cells and B cells stimulated for 48 or 96 h. At 48 h, 2.7% of the cells were IgG1-positive. The IgG1-positive and -negative fractions are shown for the 96 h time point, where the IgG1-negative fraction contained 0.5% IgG1-positive cells, and the IgG1-positive fraction contained 65%. Many cells in the IgG1-negative gate of this fraction also started to express IgG1 as they shifted in fluorescence towards the IgG1-positive gate. (TIF) [file pone.0111748.s001.tif]

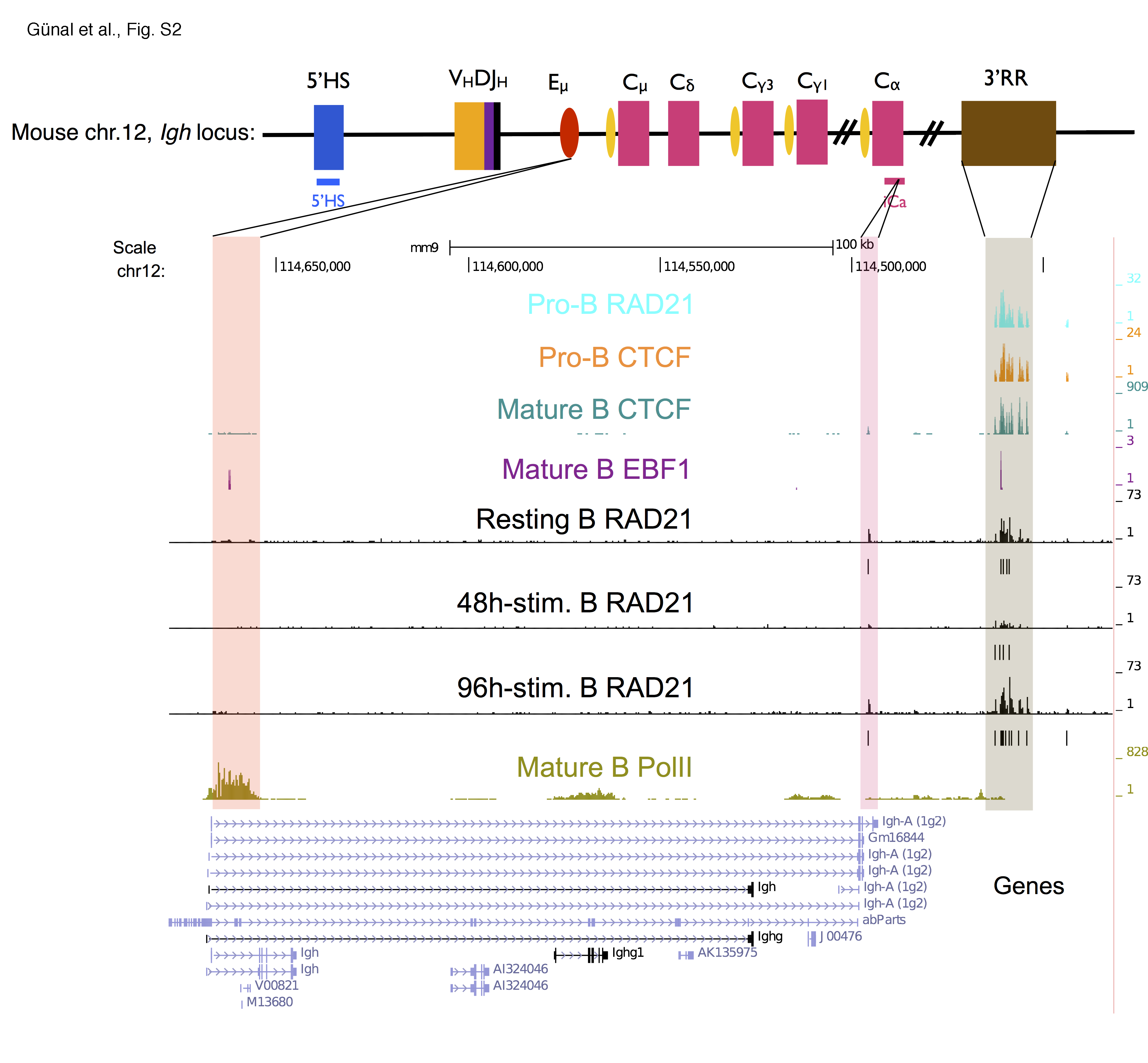

Supplement: Figure S2 — ChIP-Seq profile of binding factors around the Eμ and 3′RR. Binding profile of RAD21, CTCF, EBF1 and PolII in pro- and/or in mature B cells. The highest enrichment of PolII marks the locus enhancer Eμ. The highest cohesin-CTCF colocalization marks the cohesin/CTCF bound to 3′RR. Genes depict UCSC Gene annotations. ChIPSeq data plots are generated by using data obtained in this study and from publicly available sources mentioned in Table S1. (TIF) [file pone.0111748.s002.tif]

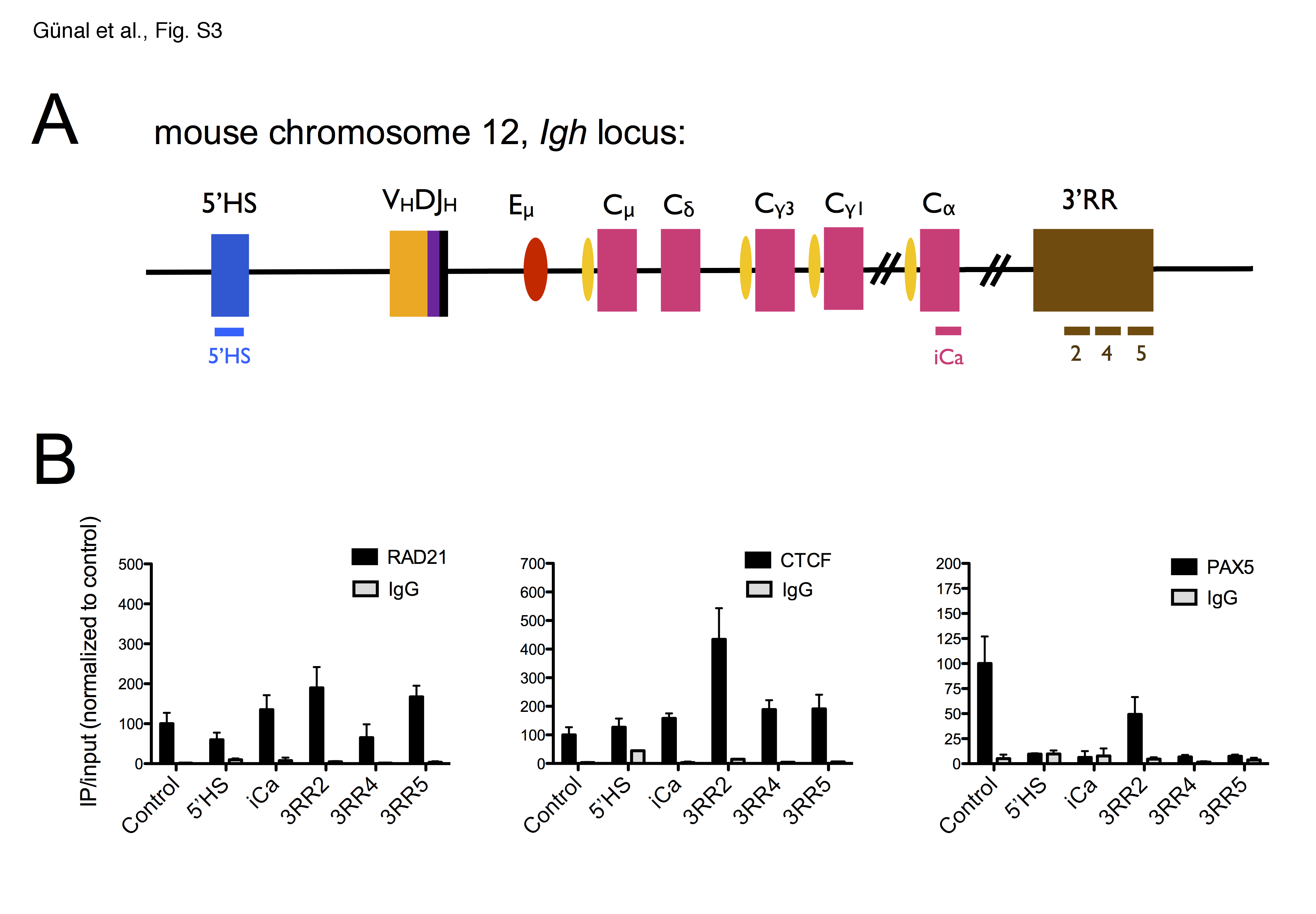

Supplement: Figure S3 — Binding of factors at Igh locus cohesin sites. (A) Scheme of the Igh locus binding events under investigation. (B) Enrichment of RAD21, CTCF, and PAX5 at cohesin binding sites in resting B cells were quantified by ChIP-qPCR. (TIF) [file pone.0111748.s003.tif]

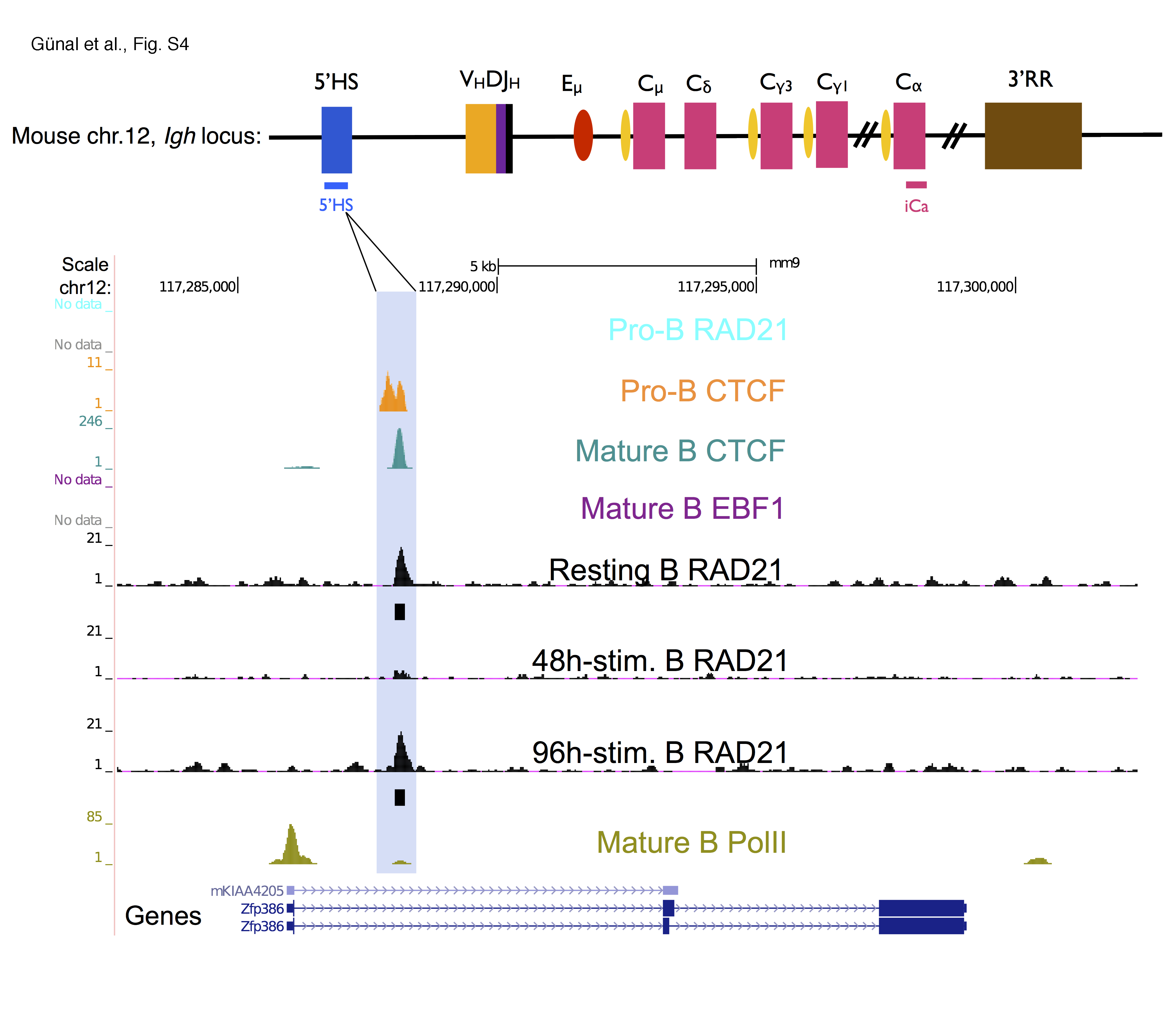

Supplement: Figure S4 — Mature B cell-specific cohesin/CTCF site corresponds to the 5′ flanking gene of Igh locus. ChIP-Seq data plot indicates a mature B cell specific binding of cohesin subunit RAD21 at the HS region located at the 5′ of Igh locus. (TIF) [file pone.0111748.s004.tif]

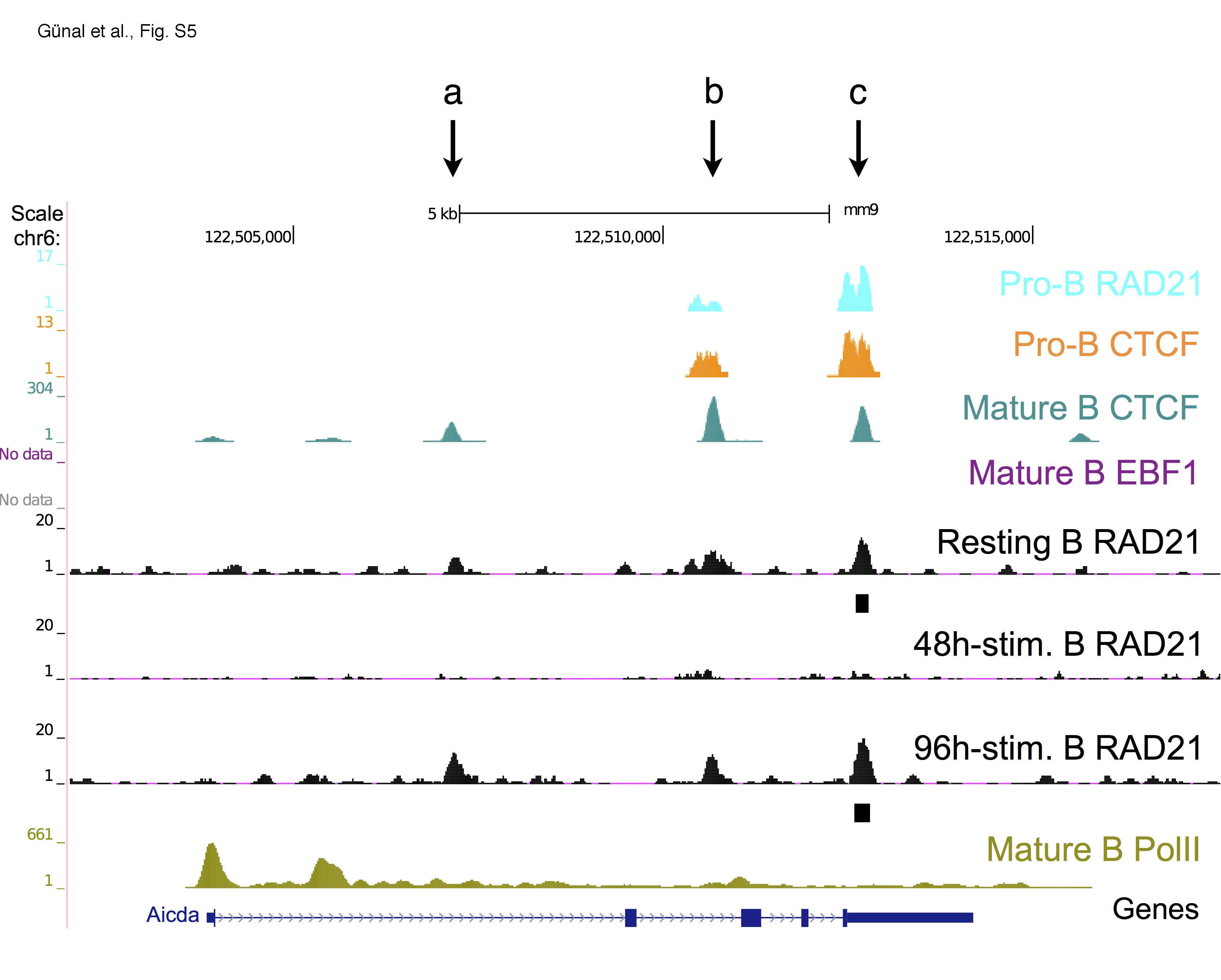

Supplement: Figure S5 — Binding profile at the Aicda gene locus. ChIP-seq profiles of RAD21, CTCF, EBF1 and PolII binding at Aicda. (TIF) [file pone.0111748.s005.tif]
