# Supplementary material for: Stage-Specific Binding Profiles of Cohesin in Resting and Activated B Lymphocytes Suggest a Role for Cohesin in Immunoglobulin Class Switching and Maturation
Source: PLoS One. 2014 Nov 6;9(11):e111748. doi: 10.1371/journal.pone.0111748 (PMC4222939; doi:10.1371/journal.pone.0111748)
Supplement: Table S1 — ChIP-seq data sets used for comparison studies. (PDF) [file pone.0111748.s006.pdf]

Table S1. ChIP-seq data sets used for comparison studies.

Factor	Mouse Background	B cell stage	Activation	Data Source
RAD21	Rag1$^{-/-}$	Pro-B	none	Degner et al, 2011 GSM644976
CTCF	Rag1$^{-/-}$	Pro-B	none	Degner et al, 2011 GSM644975
CTCF	wt	Mature B	3d with LPS+IL4	Yamane et al, 2011 GSM836458
EBF1	wt	Mature B	none	Győry et al, 2012 GSE35910
PolII	wt	Mature B	3d with LPS+IL4	Yamane et al, 2011 GSM594830
